# Sociodemographic Characteristics Associated With and Prevalence and Frequency of Cannabis Use Among Adults in the US

**DOI:** 10.1001/jamanetworkopen.2021.36571

**Published:** 2021-11-30

**Authors:** Abra M. Jeffers, Stanton Glantz, Amy Byers, Salomeh Keyhani

**Affiliations:** 1 formerly of Center for Tobacco Control Research & Education, University of California, San Francisco; 2 Center for Tobacco Control Research & Education, University of California, San Francisco; 3 Department of Psychiatry & Behavioral Sciences, University of California, San Francisco; 4 Division of Geriatrics, Department of Medicine, University of California, San Francisco; 5 Section of Mental Health Services, San Francisco Veterans Affairs Medical Center, San Francisco, California; 6 Division of Internal Medicine, Department of Medicine, University of California, San Francisco; 7 Section of General Internal Medicine, San Francisco Veterans Affairs Medical Center, San Francisco, California

## Abstract

**Importance:**

Cannabis use has increased, but there are few studies on frequent and daily cannabis use among US adults. Individuals who engage in higher frequency use may suffer more health consequences.

**Objective:**

To examine frequency of cannabis use and associated factors among US adults.

**Design, Setting, and Participants:**

This survey study included data from 21 US states and 2 US territories reported in the Behavioral Risk Factor Surveillance System surveys from 2016 to 2019. Cross-sectional data on US adults ages 18 years and older were used to estimate demographic, socioeconomic, and behavioral risk factors for cannabis use, taking into account the survey strata and sampling weights for the 4 years of combined data. Using a multivariable ordinal logistic analysis, the association of demographic, socioeconomic status, and behavioral risk factors with past month cannabis frequency were examined.

**Exposures:**

Sociodemographic characteristic, ie, age, gender, race and ethnicity, educational attainment, employment status, and annual household income.

**Main Outcomes and Measures:**

Ordinal categorization of number of days of cannabis use in the past 30 days in terms of nonuse, infrequent use (1-5 days), frequent use (6-29 days), and daily use.

**Results:**

Among the 387 179 respondents, 58 009 (27.9%) were ages 18 to 34 years, 186 923 (50.3%) were ages 35 to 64 years, and 142 225 (21.8%) were age 65 years or older (mean [SD] age, 48.3 [0.1] years). The sample included 28 345 (9.8%) Black, 36 697 (22.6%) Hispanic, and 292 210 (57.3%) White respondents. Smoking was the most common form of cannabis use. The frequency of cannabis use varied significantly by age, gender, race, marital status, education, and employment. Higher frequency cannabis use was associated with younger age (ages 18-34 years: adjusted odds ratio [aOR], 4.12; 95% CI, 3.63-4.68; ages 35-64 years: aOR, 2.22; 95% CI, 1.98-2.49), Black (aOR, 1.46; 95% CI, 1.33-1.71) and Native American (aOR, 1.25; 95% CI, 1.04-1.52) race, and less educational attainment (high school or less: aOR, 1.09; 95% CI, 1.02-1.17; some college: aOR, 1.27; 95% CI, 1.19-1.35). Being married (aOR, 0.54; 95% CI, 0.51-0.58) or identifying as Asian (aOR, 0.60; 95% CI, 0.51-0.71) or Hispanic (aOR, 0.71; 95% CI, 0.65-0.77) was associated with lower-frequency cannabis use after accounting for other baseline factors.

**Conclusions and Relevance:**

This nationally based study found that higher-frequency cannabis use is more common among young and racial minority populations, as well as respondents with low socioeconomic status. Given the known and emerging negative health effects of cannabis use, more attention may need to be paid to high-frequency use among underserved populations in the form of screening, risk stratification, and treatment.

## Key Points

**Question:**

What are the sociodemographic characteristics of adults who engage in high-frequency cannabis use?

**Findings:**

In this survey study including 387 157 US adults residing in 21 states conducted in 2016 through 2019, young, male, Black, and Native American individuals and individuals with low educational attainment and income were more likely to engage in higher frequency cannabis use.

**Meaning:**

Higher-frequency use among these populations may warrant more attention from policymakers and public health officials in the form of screening, risk stratification, and treatment given the known and emerging adverse health effects of cannabis.

## Teaser

This survey study of 21 US states and 2 US territories examines social and demographic characteristics associated with the frequency of cannabis use among adults.

## Introduction

From 2002 to 2019, past-year prevalence of cannabis use increased from 10.4% to 18.0%, while daily or almost daily use (ie, 300 or more days per year) increased from 1.3% to 3.9% among US adults.^1^ Despite the dramatic rise in past-year cannabis use and daily or almost daily use, the distribution of use frequency (ie, days per month) and determinants of higher-frequency use have not been adequately described.

Evidence for the adverse mental and physical health effects of cannabis is emerging. While the existing evidence base is insufficient and includes few studies at low risk of bias, some studies have shown that cannabis use is associated with adverse effects including respiratory symptoms (cough and sputum production), tachycardia, and some forms of cancer.^2,3,4,5,6,7,8,9,10,11,12,13,14,15,16,17,18,19^ Long-term cannabis use is associated with deficits in memory among adults.^20^ Long-term cannabis use is associated with poor educational outcomes and increased risk of psychotic disorders, although some experts believe a causal link is not yet established.^21,22,23,24,25^ Cannabis use may negatively affect mental health treatment outcomes for patients with anxiety and depression, and use is associated with suicidal ideation.^26,27,28,29,30,31,32,33,34^ Finally, recent data suggests that that 1 out of 5 individuals who use cannabis have a cannabis use disorder.^35^

Studies that have examined the health effects of cannabis have used various measures of exposure (eg, ever use, past-year use, monthly use, daily and almost daily use); however, there is a wide spectrum of outcomes for use frequency within the past month.^36^ While daily use is likely to have the most effect, like alcohol, there may be a gradient of outcomes based on frequency of use.^37^

An annual cannabis module was added to the Centers for Disease Control and Prevention’s Behavioral Risk Factor Surveillance System (BRFSS) in 2016, which allows evaluation of cannabis use patterns among adults (ages 18 and older) in 23 states and territories.^38^ We examined the distribution of frequency of use and factors associated with higher frequency use using the BRFSS survey. Understanding prevalence of cannabis use and frequency of use in different populations will shed light on groups most likely to be affected by the health consequences of use.

## Methods

### Data Source

We combined 2016 through 2019 BRFSS data from 21 American states and 2 US territories participating in the cannabis module during at least 1 of these years (eTable 1 in the Supplement). BRFSS is a telephone-administered survey that collects data from a representative sample of US adult residents regarding health-related risk behaviors, chronic conditions, and health care access.^38^ This study is based on publicly available deidentified data and is exempt from institutional review board review and informed consent requirements.

This study followed the American Association for Public Opinion Research (AAPOR) reporting guideline; response rates for BRFSS data were calculated using standards set by AAPOR response rate formula 4.^39^ The response rate is the number of respondents who completed the survey as a proportion of all eligible and likely eligible people. The median (IQR) survey response rates for all states, territories and Washington, DC, in 2016, 2017, 2018, and 2019 were 46.7% (41.2%-54.5%), 44.9% (38.6%-51.5%), 49.8% (44.2%-55.0%), and 50% (44.1%-55.3%), respectively, and ranged from 30.6% to 73.1%. Response rates for states and territories included in this analysis had a median of 49.8% and ranged from 31.1% to 65.0%. For detailed information see the BRFSS Summary Data Quality Report.^89^

### Analytic Sample

To assess cannabis use in the past month, we used the question, “During the past 30 days, on how many days did you use marijuana or hashish?” Response options included any number of days from 0 to 30, “Don’t know,” or refusing to answer the question. We excluded the 0.8% of respondents who answered “Don’t know” or refused to answer, leaving 387 157 respondents living in 21 states (ie, Alaska, California, Colorado, Florida, Georgia, Idaho, Illinois, Maryland, Minnesota, Mississippi, Montana, Nebraska, New Hampshire, North Dakota, Ohio, Oklahoma, South Carolina, Tennessee, Utah, West Virginia, Wyoming) and 2 territories (Guam, Puerto Rico) (unweighted sample sizes in Table 1). To assess the primary form of past-month cannabis use, we used the question, “During the past 30 days, which one of the following ways did you use marijuana the most often? Did you usually….” Response options included, smoke it, eat it, drink it, vaporize it (“in an e-cigarette-like vaporizer or other vaporizing device”), dab it (“using waxes or concentrates”), use it some other way, or “Don’t know/Not sure” or “Refused.” We used an answer of zero days to the question of past-month cannabis use to categorize nonuse. There were 6350 cannabis users within the past month who did not respond to the question on forms of use.

**Table 1.** Unweighted Number of Respondents by Past-Month Cannabis Use Frequency Categories Among Respondents of Behavioral Risk Factor Surveillance System Cannabis Module in Years 2016-2019

Characteristics	Respondents, No. (%)				
	Cannabis use in past month		Frequency of past-month use among cannabis users^a^		
	Nonuse	Current use	Infrequent	Frequent	Daily
Total	361 847	25 310	9904	6951	8455
Age, y					
18-34	48 842	9167	3447	2641	3079
35-64	174 051	12 872	5052	3379	4441
>65	138 954	3271	1405	931	935
Sex					
Men	156 023	15 348	5727	4303	5318
Women	205 696	9951	4176	2643	3132
Race and ethnicity					
African American	26 170	2175	847	489	839
Asian, Native Hawaiian, or Pacific Islander	8959	623	334	148	141
Hispanic	34 112	2585	1032	701	852
Native American	4916	693	207	172	314
White	274 435	17 775	6987	5049	5739
Other	7379	1022	349	274	399
Marital status					
Not married	164 301	16 852	6356	4636	5860
Married	195 508	8333	3508	2273	2552
Education					
≤High school degree	125 669	9448	3229	2363	3856
Some college	101 287	8389	3203	2397	2789
College degree	133 840	7440	3461	2178	1801
Employment					
Employed	175 107	14 769	5899	4004	4866
Unemployed	37 706	4932	1622	1296	2014
Student	7809	1372	670	425	277
Retiree	118 416	3387	1396	979	1012
Homemaker	20 598	726	272	205	249
Annual household income, $					
<25 000	79 775	7478	2625	1966	2887
25 000 to <75 000	124 534	6469	3338	24,53	3131
≥75 000	102 364	6271	2909	1829	1533
Legality of cannabis in state of residence					
Nonlegal	137 605	6761	2742	1795	2224
Legalized cannabis					
Medical	185 194	12 721	4943	3436	4342
Recreational	39 048	5828	2219	1720	1889
Smoker					
Current	45 074	9416	2989	2515	3912
Former	101 291	7412	2808	2074	2530
Never	213 411	8364	4056	2328	1980
e-Cigarette use					
Current	6770	2327	718	671	938
Former	28 575	6919	2552	1945	2422
Never	222 086	7557	3433	1971	2153
No e-cigarette question^b^	83 460	7373	2757	2078	2538
Alcohol					
Any alcohol in past 30 d	172 702	18 163	7637	5161	5365
Any alcohol binges in past 30 d	39 008	9294	3827	2791	2676

^a^ Infrequent use was defined as 1 to 5 days of cannabis use in the past month; frequent, as 6 to 29 days; and daily, as 30 days of use in the past month.

^b^ The 2019 survey did not include a question about e-cigarette use.

### Independent Variables

Demographic variables were age, gender, marital status, and self-identified race and ethnicity. Race and ethnicity were coded mutually exclusively as Hispanic, non-Hispanic Asian and Pacific Islander, non-Hispanic Black, non-Hispanic Native American or Alaskan Native, non-Hispanic White, and other.

Socioeconomic status was represented by educational attainment, employment status, and annual household income. Educational attainment was categorized by combining 4 categories corresponding to high school degree or less, some college, and college degree. Employment status was divided into employed, unemployed, student, retired, and homemaker categories. The educational and employment categories were defined by BRFSS. Annual household income was divided into less than $25 000, $25 000 to $74 999, and $75 000 or higher categories corresponding to 2019 federal annual thresholds for family of 4 poverty and median income.^40,41^ Legality of cannabis in state of residence for an individual was determined by their year of response to the BRFSS survey and divided into 3 separate categories: nonlegal cannabis, legalized medical cannabis, and legalized recreational cannabis (eTable 1 in the Supplement). All states that had legalized recreational cannabis also had legalized medical cannabis.

We controlled for differences in the behavioral risk factor domain of current, former, and nonuse of cigarettes and e-cigarettes, current alcohol use, and current binge drinking by including these covariates in the model. People who engaged in past-month binge drinking were a subset of past-month users of alcohol, where binge drinking was defined as responding to the following question with any number greater than zero, “Considering all types of alcoholic beverages, how many times during the past 30 days did you have 5 or more drinks for men or 4 or more drinks for women on an occasion?”

### Outcome

We first categorized persons who use cannabis into daily and nondaily use and nonuse. To create a more granular distribution of cannabis use frequency, we divided nondaily use into infrequent and frequent nondaily use based on the median number of days of nondaily use (5 days). Thus, we categorized frequency of cannabis use into 4 categories: nonuse, infrequent (1-5 days), frequent (6-29 days), and daily cannabis use in the past 30 days.

### Statistical Analysis

Weighted estimates of demographic, socioeconomic, and behavioral risk factors were calculated using survey strata and sampling weights for the 4 years of combined data to obtain representative results for the combined states using the cannabis module.^42^* P* values for bivariate analyses were calculated by the Rao-Scott corrected χ^2^ test.

We conducted a multivariable ordinal logistic analysis of the association of past-month cannabis use ordinal frequency category (nonuse, infrequent, frequent, and daily use) as a function of demographic, socioeconomic status, and behavioral risk factors, accounting for the complex survey design of BRFSS. The proportional odds ratio (OR) assumption of an ordinal regression assumes that the associations between the highest vs all lower categories of the outcome variable are the same as those that describe the associations between the next highest category and all lower categories.^43^ We statistically and graphically tested the proportional OR assumption and the assumption was met. An OR greater than 1 for a certain variable implied that this variable was associated with higher frequency of use than the reference category, while an OR less than 1 implied that this variable was associated with a lower frequency of cannabis use.

Income had the highest degree of missingness, with 14.9% of the variables used in the ordinal regression. Listwise deletion of all cases with any missing variables resulted in data loss of 22.6% of the sample, necessitating multiple imputation.^44^ The multivariable ordinal logistic analysis was conducted using 23 multiple imputations with chained equations using the R statistical software version 4.0 (R Project for Statistical Computing) amelia package.^45^ Statistics were performed using the R survey package.

We conducted 2 sensitivity analyses to examine the robustness of our findings: (1) multivariable ordinal logistic regression in the set of complete cases was used to examine whether findings of the analyses employing imputed data and complete cases were similar; (2) because the 2 US territories (Guam and Puerto Rico) might affect ordinal logistic regression estimates on race and ethnicity, we excluded respondents from the 2 territories.

## Results

Among the 387 179 respondents to the cannabis module, the prevalence of cannabis nonuse was 90.0% (361 847 respondents), infrequent cannabis use (ie, 1 to 5 days; median [IQR] use, 2 [1-3] days) was 3.7% (9904 respondents), frequent use (6 to 29 days; median [IQR] use, 15 [10-20] days) was 2.8% (6951 respondents), and daily use was 3.5% (8455 respondents).

The mean (SD) age of the respondents was 48.3 (0.1) years. Approximately 27.9% of individuals were ages 18 to 34 years in our weighted sample estimate, 50.3% were ages 35 to 64 years, and 21.8% were over age 65 years. More than half (51.5%) were women and the majority of the respondents were White (57.3%), 7.6% Asian, 9.8% were Black, 22.6% Hispanic, 0.8% Native American, and 1.9% other. About 40.9% had a high school education or less, and 27.2% had a college degree. Most of the sample were never smokers (61.4%), 24.2% had formerly smoked tobacco, and 14.4% currently smoked tobacco. Similarly, 59.3% of respondents were persons who had never used e-cigarettes, 12.4% were persons who formerly used e-cigarettes, 3.2% currently used e-cigarettes, and 25.1% of the sample were not asked a question about e-cigarette use in 2019. Over half of the sample consumed at least 1 alcoholic beverage in the past 30 days, while 15.6% had engaged in binge drinking within the past month.

### Variation in the Prevalence and Frequency of Cannabis Use Among Different Sociodemographic Groups

Among respondents, 10.1% (95% CI, 9.8%-10.2%) used cannabis in the past month, including 3.8% (95% CI, 3.6%-3.8%) with infrequent use, 2.8% (95% CI, 2.7%-2.9%) with frequent use, and 3.5% (95% CI, 3.4%-3.7%) with daily use (Table 2). Smoking was the most common form of cannabis use (5.5%), followed by vaping (0.9%) and edibles (0.8%) (eFigure 1 in the Supplement). Daily use was more common among individuals who used smoked cannabis, while infrequent use was more common among those using edible cannabis (eFigure 1 in the Supplement)

**Table 2.** Past-Month Cannabis Use Frequency Categories Among Respondents of Behavioral Risk Factor Surveillance System Cannabis Module Between Years 2016 and 2019^a^

Covariates	Respondents, weighted % (95% CI)					*P* value^c^
	Past-month cannabis use		Frequency of past-month use among cannabis users^b^			
	Nonusers	Current users	Infrequent	Frequent	Daily	
Total	90.0 (89.8-90.2)	10.0 (9.8-10.2)	3.8 (3.6-3.8)	2.8 (2.7-2.9)	3.5 (3.4-3.7)	
Age, y						
18-34	25.3 (25.0-25.7)	49.9 (48.7-51.1)	48.3 (46.4-50.1)	51.9 (49.7-54.1)	50.1 (48.0-52.1)	<.001
35-64	51.1 (50.7-51.4)	43.3 (42.1-44.4)	44.1 (42.3-46.0)	41.0 (38.8-43.1)	44.2 (42.2-46.2)	
≥65	23.6 (23.3-23.9)	6.8 (6.3-7.3)	7.6 (6.8-8.4)	7.1 (6.2-8.1)	5.7 (4.0-6.5)	
Sex						
Men	46.7 (46.4-47.1)	61.6 (60.5-62.8)	57.8 (56.0-59.7)	62.2 (60.0-64.3)	65.2 (63.3-67.2)	<.001
Women	53.3 (52.9-53.6)	38.4 (37.2-39.5)	42.2 (40.3-44.0)	37.8 (35,7-40.0)	34.8 (32.8-36.7)	
Race and ethnicity						
African American	11.2 (11.0-11.5)	14.1 (13.1-15.0)	12.5 (11.1-13.8)	12.9 (11.1-14.7)	16.8 (15.0-18.6)	<.001
Asian, Native Hawaiian, or Pacific Islander	6.1 (5.8-6.3)	4.3 (3.7-4.9)	6.0 (4.8-7.1)	4.3 (3.2-5.3)	2.7 (1.9-3.5)	
Hispanic	20.4 (20.1-20.7)	17.9 (16.9-18.8)	19.0 (17.4-20.6)	17.3 (15.6-19.0)	17.2 (15.5-18.8)	
Native American	0.8 (0.8-0.9)	1.3 (1.1-1.5)	1.0 (0.7-1.2)	1.1 (0.8-1.4)	1.8 (1.6-1.9)	
White	59.8 (59.5-60.2)	59.5 (58.4-60.8)	59.0 (57.1-60.9)	61.9 (59.6-64.1)	58.4 (56.3-60.5)	
Other	1.7 (1.6-1.7)	2.8 (2.5-3.1)	2.6 (2.2-3.1)	2.5 (2.0-3.2)	3.2 (2.7-3.7)	
Marital status						
Not married	46.9 (46.6-47.3)	70.5 (69.5-71.6)	68.8 (67.1-70.5)	70.0 (68.0-72.1)	72.7 (70.9-74.5)	<.001
Married	53.1 (52.7-53.4)	29.5 (28.3-30.5)	31.2 (29.5-32.3)	30.0 (27.9-32.0)	27.3 (25.5-29.1)	
Education						
≤High school degree	41.1 (40.8-41.5)	41.3 (40.1-42.5)	35.8 (34.0-37.6)	36.7 (34.6-38.9)	50.7 (48.6-52.3)	<.001
Some college	31.0 (30.6-31.3)	38.7 (37.6-39.9)	39.1 (37.2-41.0)	41.5 (39.3-43.8)	36.2 (34.2-38.2)	
College degree	27.9 (27.6-28.2)	20.0 (10.2-20.7)	25.1 (23.6-26.5)	21.7 (20.2-23.2)	13.2 (12.1-14.3)	
Employment						
Employed	55.7 (55.3-56.0)	63.4 (62.6-64.8)	63.2 (61.4-65.0)	63.5 (61.4-65.6)	64.3 (62.4-66.2)	<.001
Unemployed	11.5 (11.3-11.8)	16.9 (16.1-17.7)	14.3 (13.1-15.5)	16.3 (14.7-17.9)	20.1 (18.5-21.6)	
Student	4.9 (4.7-5.1)	8.8 (8.1-9.5)	11.4 (10.1-12.7)	9.2 (7.9-10.5)	5.7 (4.6-6.9)	
Retiree	20.8 (20.6-21.1)	7.7 (7.2-8.3)	8.0 (7.1-8.9)	8.0 (7.1-9.0)	7.2 (6.3-8.1)	
Homemaker	7.0 (6.9-7.2)	2.9 (2.5-3.3)	3.1 (2.5-3.8)	2.9 (2.2-3.6)	2.7 (2.1-3.3)	
Annual household income, $						
<25 000	27.9 (27.6-28.3)	30.4 (29.3-31.5)	26.8 (25.2-28.5)	29.1 (27.1-31.2)	35.1 (33.1-37.2)	<.001
25 000 to <75 000	37.6 (37.2-37.9)	37.3 (36.1-38.5)	36.1 (34.2-38.0)	36.3 (34.1-38.5)	39.3 (37.1-41.4)	
≥75 000	34.5 (34.2-34.9)	32.3 (31.1-33.5)	37.1 (35.1-39.0)	34.5 (32.3-36.8)	25.6 (23.5-27.6)	
Legality of cannabis in state of residence						
Nonlegal	32.5 (32.3-32.8)	22.9 (22.0-23.9)	22.9 (21.4-24.4)	22.2 (20.3-24.1)	23.6 (21.9-25.3)	<.001
Legalized cannabis						
Medical	43.9 (43.7-44.2)	41.7 (40.6-42.9)	41.6 (39.8-43.5)	40.1 (38.0-42.3)	43.1 (41.0-45.1)	
Recreational	23.5 (23.3-23.8)	35.3 (34.6-36.4)	35.4 (33.7-37.2)	37.7 (35.6-39.8)	33.3 (31.5-35.2)	
Smoker						
Current	13.0 (12.7-13.2)	34.5 (33.3-35.6)	26.5 (24.8-28.1)	33.7 (31.6-35.8)	43.5 (41.4-45.5)	<.001
Former	24.0 (23.7-24.3)	26.1 (25.1-27.1)	24.5 (23.0-26.1)	26.6 (24.7-28.5)	27.3 (25.5-29.0)	
Never	63.0 (62.7-63.3)	39.5 (38.3-40.6)	49.0 (47.1-50.9)	39.7 (37.6-41.9)	29.3 (27.3-31.2)	
e-Cigarettes						
Current	2.5 (2.4-2.6)	10.5 (9.7-11.2)	8.5 (7.4-9.5)	10.5 (9.1-12.0)	12.6 (11.1-14.0)	<.001
Former	10.1 (9.9-10.3)	30.3 (29.2-31.4)	28.9 (27.1-30.6)	31.5 (29.4-33.7)	30.8 (28.9-32.6)	
Never	61.3 (61.0-61.6)	27.4 (26.4-28.4)	32.4 (30.7-34.2)	25.6 (23.7-27.4)	23.5 (21.8-25.2)	
No e-cigarette question^d^	26.1 (25.9-26.4)	31.9 (30.7-33.0)	30.2 (28.5-32.0)	32.4 (30.2-34.5)	33.2 (31.1-35.3)	
Alcohol						
Any alcohol in past 30 d	48.5 (48.2-48.9)	75.3 (74.3-76.3)	78.5 (76.9-80.1)	77.3 (75.4-79.2)	70.4 (68.6-72.1)	<.001
Any alcohol binges in past 30 d	12.8 (12.5-13.0)	39.7 (38.5-40.8)	39.8 (37.9-41.6)	43.9 (41.7-46.2)	36.2 (34.1-38.3)	<.001

^a^ The cannabis module was administered in: Alaska, California, Colorado, Florida, Georgia, Idaho, Illinois, Maryland, Minnesota, Mississippi, Montana, North Dakota, Nebraska, New Hampshire, Ohio, Oklahoma, South Carolina, Tennessee, Utah, West Virginia, Wyoming, Guam, and Puerto Rico. Idaho, Minnesota, Tennessee, and Wyoming were the 4 states participating in the cannabis module across all 4 years of the combined 2016-2019 BRFSS data. See eTable 1 in the Supplement.

^b^ Infrequent use was defined as 1 to 5 days of cannabis use in the past month; frequent, as 6 to 29 days; and daily, as 30 days of use in the past month.

^c^ *P* value for the null hypothesis that cannabis use frequency does not vary by covariate of interest.

^d^ The 2019 survey did not include a question about e-cigarette use.

Cannabis use varied significantly by age, gender, race, marital status, education, employment, and income (Table 2). Infrequent (weighted percentage, 6.4%), frequent (5.2%), and daily use (6.3%) were more prevalent among the youngest age group (18 to 34 years old) compared with the oldest age group (≥65 years) (infrequent, 1.3%; frequent, 0.9%; daily, 0.9%) (eFigure 2 in the Supplement). Cannabis use was more prevalent among men (infrequent, 4.4%; frequent, 3.6%; daily, 4.8%) than women (infrequent, 3.0%; frequent, 2.0%; daily, 2.4%). There was significant variation among racial and ethnic groups. Black (5.1%) and Native American (7.2%) cannabis users were more likely to be daily users, while Asian cannabis consumers tended to be infrequent users.

Unmarried individuals engaged in higher frequency use (infrequent, 5.2%; frequent, 3.9%; daily, 5.2%) compared with married individuals (infrequent, 2.3%; frequent, 1.6%; daily, 1.9%) (eFigure 3 in the Supplement). Daily cannabis use was more common among individuals with high school education or less (infrequent, 3.2%; frequent, 2.5%; daily, 4.3%) compared with individuals with college degrees (infrequent, 3.4%; frequent, 2.2%; daily, 1.7%) (eFigure 4 in the Supplement). The prevalence of daily cannabis use was lower for those with higher annual income (<$25 000, 4.6%; $25 000-$74 999, 3.8%; ≥$75 000, 2.7%).

### Dual Use of Cannabis With Tobacco and Alcohol

The prevalence of the dual use of cannabis and smoked tobacco was 3.4% (34.5% of cannabis users), while the prevalence of the dual use of cannabis and e-cigarettes was 1.4% (15.4% of persons who use cannabis) and the prevalence of the dual use of cannabis and binge drinking was 3.9% (39.7% of persons who use cannabis) (eFigure 5 in the Supplement). Over two-thirds of dual users of cannabis and tobacco (71.5%) or e-cigarettes (70.1%) were daily or frequent cannabis users. Over half (62.8%) of dual binge drinkers and cannabis users engaged in frequent and daily use.

Cannabis use was common among people that engaged in smoking tobacco (infrequent, 6.5%; frequent, 6.2%; daily, 10.1%) compared with persons that formerly smoked tobacco (infrequent, 3.8%; frequent, 3.0%; daily; 4.0%,) and never users of smoked tobacco (infrequent, 3.0%; frequent, 1.8%; daily; 1.7%) (eFigure 6 in the Supplement). Frequent and daily cannabis use was more prevalent among persons who currently (infrequent, 9.6%; frequent, 9.0%; daily, 13.6%) and formerly used e-cigarettes (infrequent, 8.9%; frequent, 7.3%; daily, 9.0%) compared with those who have never used e-cigarettes (infrequent, 2.1%; frequent, 1.2%; daily, 1.4%). Prevalence of infrequent, frequent, and daily use was higher among persons who engaged in binge drinking (infrequent, 9.5%; frequent, 7.9%; daily, 8.2%) than those who did not (infrequent, 2.6%; frequent, 1.8%; daily, 2.6%) (eFigure 7 in the Supplement)

### Variation in Higher-Frequency Cannabis Use Among States With Different Legal Status

Prevalence of cannabis use differed significantly by state cannabis legality. Prevalence of infrequent (5.3%), frequent (4.2%), and daily use (4.7%) of cannabis was higher in those states with recreational legal cannabis than other states with nonlegal use (infrequent, 2.7%; frequent, 1.9%; daily, 2.6%) or medically legal use (infrequent, 3.5%; frequent, 2.5%; daily, 3.5%) (eFigure 8 in the Supplement).

### Multivariate Ordinal Regression Determination of Factors Associated With Frequency of Cannabis Use

Compared with respondents ages 65 years or older, younger individuals were more likely to engage in higher-frequency cannabis use (ages 18-34 years: adjusted OR [aOR], 4.26; 95% CI, 3.88-4.68; ages 35-64 years: aOR, 2.33; 95% CI, 2.14-2.54) (Figure). Men were more likely to use cannabis at higher frequencies than women (aOR, 1.43; 95% CI, 1.35-1.51). Compared with White respondents, Black respondents (aOR, 1.48; 95% CI, 1.35-1.62) and Native American respondents (aOR, 1.28; 95% CI, 1.06-1.55) used cannabis at higher frequency, while Asian respondents (aOR, 0.56; 95% CI, 0.47-0.66) and Hispanic respondents (aOR, 0.65; 95% CI, 0.60-0.71) used cannabis with less frequency.

**Figure.**
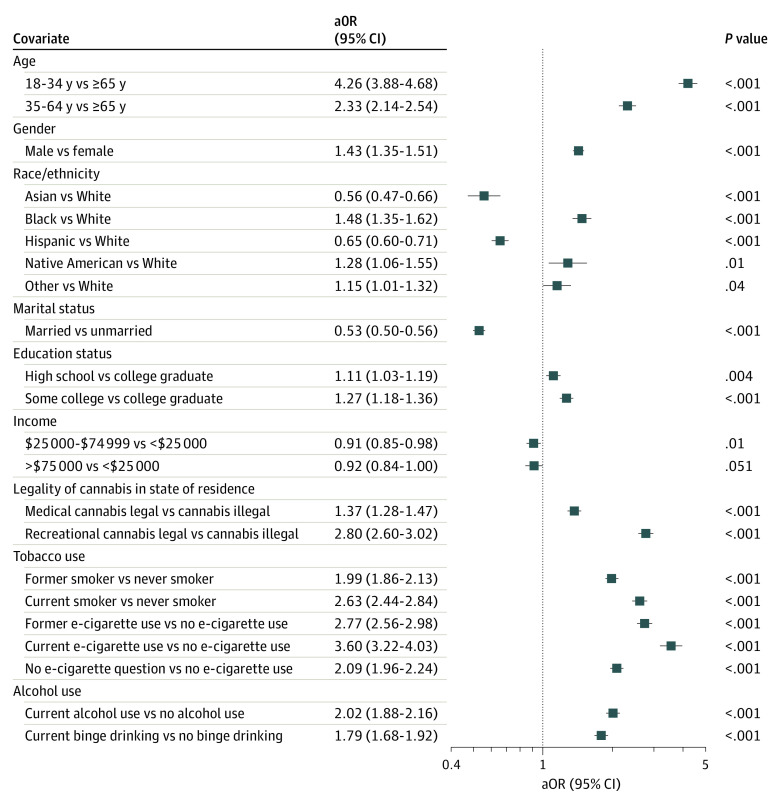
Adjusted ORs for Increased Cannabis Use Frequency Using Multiple Imputation Among Behavioral Risk Factor Surveillance System Respondents to the Cannabis Module, 2016-2019 aOR indicates adjusted odds ratio.

Married respondents were less likely to use cannabis at higher frequencies compared with unmarried individuals (aOR, 0.53; 95% CI, 0.50-0.56). Compared with individuals with a college degree, having only a high school diploma or less (aOR, 1.11; 95% CI, 1.03-1.19) or some college experience (aOR, 1.27; 95% CI, 1.18-1.36) was associated with higher frequency cannabis use. Compared with the respondents with annual household incomes below $25 000, those with incomes between $25 000 and $74 999 (aOR, 0.91; 95% CI, 0.85-0.98) used cannabis with lower frequency, while those with incomes $75 000 and above were not significantly different (aOR, 0.92; 95% CI, 0.84-1.00).

Persons who currently (aOR, 2.63; 95% CI, 2.44-2.84) or formerly (aOR, 1.99; 95% CI, 1.86-2.13) smoked tobacco engaged in higher frequency cannabis use compared with never tobacco smokers. Current and former history of e-cigarette use was associated with higher frequency cannabis use (current: aOR, 3.60; 95% CI, 3.22-4.03; former: aOR, 2.77; 95% CI, 2.56-2.98) compared with nonuse of e-cigarettes. Alcohol use in the past 30 days (aOR, 2.02; 95% CI, 1.88-2.16) and binge drinking in the past 30 days (aOR, 1.79; 95% CI, 1.68-1.92) were associated with higher-frequency cannabis use. Compared with states where no cannabis use was legal, residence in a state with medically legal (aOR, 1.37; 95% CI, 1.28-1.47) or recreationally legal (aOR, 2.80; 95% CI, 2.60-3.02) cannabis use was associated with higher-frequency cannabis use.

### Sensitivity Analyses

There were no substantial differences between the complete case analysis and that with multiple imputation except for the OR of high school education vs college degree, which was no longer significant. Excluding the 2 US territories from the analysis also resulted in no substantial change in the association between baseline factors and cannabis use frequency. We also compared the prevalence of model variables within sample (states during years where optional BRFSS cannabis module was given) and out of sample (states during years when optional cannabis module was not given) (eTable 2 in the Supplement). In-sample and out-of-sample covariates were distributed significantly differently at the 95% confidence level with the exceptions of differences for gender and marital status, which were not significantly different. Respondents to BRFSS in states that did not ask the cannabis module were younger and more likely to identify as White or Black, with a lower proportion of Hispanic individuals (eTable 2 in the Supplement). They also had greater prevalence of tobacco and alcohol use and less access to legalized recreational cannabis but greater access to medical cannabis. However, although there were statistically significant differences, most quantifiable differences between sample characteristics were quite small (eg, covariates for respondents aged 18-34 years: in sample, 27.9% vs out of sample, 29.7%; *P* < .001).

## Discussion

Most research examining the association of cannabis use with demographic and behavioral factors have been studies with high risk of bias. Moreover, it has focused on past-month or past-year cannabis use, which does not inform our understanding of populations most likely to be affected by the health consequences of use. Some studies have looked at daily use, but these assume that nondaily users are homogeneous in risk, whereas they are likely heterogeneous given the potential spectrum of use frequency among nondaily users.^46,47,48^ We derived a data-driven categorization of past-month use based on the 5 days per month median of nondaily use. Infrequent use was more prevalent among students and married individuals. Both frequent and daily use were more common among younger individuals and men. Individuals who engaged in daily use were disproportionately Black or Native American and with lower socioeconomic status.

Legalization of cannabis has been partly driven by advocacy around the racial disparities in the legal system on possession and use of cannabis, with the goal of replacing a criminal justice approach to harm reduction.^49^ Our data demonstrates that higher-frequency cannabis use is more common among the same individuals that were the targets of the criminal justice system in terms of unfair sentencing (ie, young Black men).^50^ Higher-frequency cannabis use in these populations is especially a cause for concern, given the existing and emerging research that demonstrates the harms from use increase with frequency of use^51,52,53^

Our analysis also demonstrated that higher frequency cannabis use is concentrated among younger adults. The period between ages of 18 to 34 years are a crucial time for career development and higher education. Cannabis use, in particular use at least 4 days per week, is associated with neurocognitive deficits and poor educational and other social outcomes, especially in adolescents and young adults.^51,54,55^ The prevalence of cannabis use disorder is currently low in the US population (1.7%); however, it is underdiagnosed, and more cannabis use is associated with the development of cannabis use disorder.^1,32,56^ We found that over 6.4% of US (1 out of 16) adults engaged in frequent or daily use. These data suggest that young, Black, and Native American individuals may be more likely to be at risk for cannabis use disorder given that daily use is more common in this population. It is also possible that many young adults with higher frequency cannabis use are currently not being identified and screened for use disorders. Current guidelines only recommend screening for illicit drug use (including cannabis) in primary care when accurate diagnosis and treatment of substance use disorders are available in primary care or referral.^57^ However, there has been little research on how to practically diagnose and treat primary care patients with cannabis use disorders, and many primary care clinicians feel they lack the skills, time, and support necessary to diagnose and treat substance use disorders.^58,59,60,61^

Legalization of recreational cannabis has led to increased cannabis use.^62^ We found that higher frequency cannabis use was also more common among recreationally legal states. Because legalization has not prioritized public health, residents of US states with recreationally legal cannabis have higher exposure to cannabis advertising, media messages on benefits of cannabis, cannabis home delivery, and widespread dispensaries, which may perpetuate higher frequency of use.^63,64,65,66,67,68,69,70,71,72,73^ While state leaders have promoted the tax and criminal justice benefits of recreational legalization, the association of higher-frequency use with legalized recreational use may also eventually affect young adults from racial minority groups in those states (in the form of cannabis use disorders) and possibly the health systems (in the form of higher health care costs).^74,75^ While there are little data on the association of cannabis use with health care costs, emerging evidence indicates cannabis use is associated with motor vehicle accidents, increased poison control calls, and emergency department visits, suggesting that health system costs are possible.^76,77,78,79,80,81,82,83^

Cannabis use was also common among individuals with other adverse health behaviors, such as tobacco and alcohol use, suggesting that dual use with other legal substances may become an important public health consideration. Both cannabis and alcohol have psychoactive effects and are associated with impaired driving.^84^ In combination with alcohol, cannabis has a significantly enhanced impact on cognition, attention, and concentration and can potentiate the effects of impairment.^85,86^ Rates of driving under the influence of both alcohol and cannabis have risen in states with cannabis legalization.^87^ Given that high-frequency cannabis use is more common in recreationally legal states, more public health attention and campaigns on the dangers of combined cannabis and alcohol use in these states may be warranted. We also found that higher-frequency cannabis use was common among persons who use tobacco, including e-cigarettes. There is a limited literature examining the association of the dual use of cannabis and tobacco with health, but given that smoking is the most common form of cannabis use, the combination of tobacco use (either smoked or through e-cigarettes) and cannabis on cancer and cardiovascular and respiratory health is an important future direction for research.^88^ While our study has not examined any adverse or positive outcomes associated with cannabis use or high-frequency cannabis use, it suggests that with large enough samples, the association of high-frequency cannabis use and outcomes should be investigated.

### Limitations

There are limitations to our study. Our sample may not be nationally representative because it only included the 21 states that were administered in the BRFSS cannabis module. While there were statistically significant differences among states that participated in the cannabis module and those that did not, most quantifiable differences between sample characteristics were small (eTable 2 in the Supplement). While respondents in states with legalized medical cannabis used cannabis on more days, we do not know to what extent, if any, this is the result of advice by health care professionals to do so. There were also no data on how many times per day respondents were using cannabis or the total amount of cannabis consumed. In the current study we were unable to measure the amount of use, patterns of use over time, or existing cannabis use disorder, and we did not examine other cannabis-associated problems.

## Conclusions

This study of BRFSS data from 21 US states and 2 territories found that higher-frequency cannabis use was concentrated among younger and male adults, as well as adults who identified as Black or Native American or were from communities with low socioeconomic status. The decriminalization and legalization of cannabis was partly driven by social and racial justice concerns. Higher-frequency use among individuals in younger and racial minority populations is a cause for concern and may warrant attention from policymakers and public health officials. Furthermore, more attention may need to be paid to high frequency use in the form of screening, risk stratification, and treatment given the known and emerging negative health effects of cannabis.

## Notes

Supplement. **eTable 1.** Data Availability by State and Year, Color-Coded by Legality of Cannabis **eTable 2.** Distribution of Covariates Within Sample (States During Years Where Optional Cannabis Module Was Given, n = 387 157) and Out of Sample (States During Years When Optional Cannabis Module Was Not Given, n = 1 404 866) **eFigure 1.** Prevalence of Prominent Forms of Cannabis Use by Frequency of Use **eFigure 2.** Prevalence of Cannabis Use Frequency by Age Group, Gender, and Race/Ethnicity **eFigure 3.** Prevalence of Cannabis Use Frequency by Marital Status **eFigure 4.** Prevalence of Cannabis Use Frequency by Educational Attainment, Employment Status, and Annual Income **eFigure 5.** Prevalence of Dual Use of Cannabis and Smoked Tobacco, e-Cigarettes, and Binge Drinking by Frequency of Cannabis Use **eFigure 6.** Prevalence of Cannabis Use Frequency by Tobacco Product Use **eFigure 7.** Prevalence of Cannabis Use Frequency by Alcohol Use **eFigure 8.** Prevalence of Cannabis Use Frequency by Legality of Cannabis in State of Residence
