# Supplementary material for: Sociodemographic Characteristics Associated With and Prevalence and Frequency of Cannabis Use Among Adults in the US
Source: JAMA Netw Open. 2021 Nov 30;4(11):e2136571. doi: 10.1001/jamanetworkopen.2021.36571 (PMC8634054; doi:10.1001/jamanetworkopen.2021.36571)
Supplement: Supplement. — eTable 1. Data Availability by State and Year, Color-Coded by Legality of Cannabis eTable 2. Distribution of Covariates Within Sample (States During Years Where Optional Cannabis Module Was Given, n = 387 157) and Out of Sample (States During Years When Optional Cannabis Module Was Not Given, n = 1 404 866) eFigure 1. Prevalence of Prominent Forms of Cannabis Use by Frequency of Use eFigure 2. Prevalence of Cannabis Use Frequency by Age Group, Gender, and Race/Ethnicity eFigure 3. Prevalence of Cannabis Use Frequency by Marital Status eFigure 4. Prevalence of Cannabis Use Frequency by Educational Attainment, Employment Status, and Annual Income eFigure 5. Prevalence of Dual Use of Cannabis and Smoked Tobacco, e-Cigarettes, and Binge Drinking by Frequency of Cannabis Use eFigure 6. Prevalence of Cannabis Use Frequency by Tobacco Product Use eFigure 7. Prevalence of Cannabis Use Frequency by Alcohol Use eFigure 8. Prevalence of Cannabis Use Frequency by Legality of Cannabis in State of Residence [file jamanetwopen-e2136571-s001.pdf]

Supplemental Online Content

Jeffers AM, Glantz S, Byers A, Keyhani S. Sociodemographic characteristics associated with and prevalence and frequency of cannabis use among adults in the US. *JAMA Netw Open.* 2021;4(11):e2136571. doi:10.1001/jamanetworkopen.2021.36571

eTable 1. Data Availability by State and Year, Color-Coded by Legality of Cannabis

eTable 2. Distribution of Covariates Within Sample (States During Years Where Optional Cannabis Module Was Given, N = 387 157) and Out of Sample (States During Years When Optional Cannabis Module Was Not Given, N = 1 404 866)

eFigure 1. Prevalence of Prominent Forms of Cannabis Use by Frequency of Use

eFigure 2. Prevalence of Cannabis Use Frequency by Age Group, Gender, and Race/Ethnicity

eFigure 3. Prevalence of Cannabis Use Frequency by Marital Status

eFigure 4. Prevalence of Cannabis Use Frequency by Educational Attainment, Employment Status, and Annual Income

eFigure 5. Prevalence of Dual Use of Cannabis and Smoked Tobacco, e-Cigarettes, and Binge Drinking by Frequency of Cannabis Use

eFigure 6. Prevalence of Cannabis Use Frequency by Tobacco Product Use

eFigure 7. Prevalence of Cannabis Use Frequency by Alcohol Use

eFigure 8. Prevalence of Cannabis Use Frequency by Legality of Cannabis in State of Residence

This supplemental material has been provided by the authors to give readers additional information about their work.

© 2021 Jeffers AM et al. *JAMA Network Open.*

eTable 1. Data Availability by State and Year, Color-Coded by Legality of Cannabis. Red means illegal cannabis, light green is legal medical cannabis only, and dark green is legal recreational and medical cannabis. Respondents to BRFSS cannabis module in a given year were assigned the cannabis legal status of the state in that year.

State	Year			
	2016	2017	2018	2019
Alaska	R	R	--[a]	--
California	M	R	R	R
Colorado	R	--	--	--
Florida	I	--	M	--
Georgia	--	I	--	--
Guam	--	M	M	R
Idaho	I	I	I	I
Illinois	--	--	--	M
Maryland	--	--	M	M
Minnesota	M	M	M	M
Mississippi	I	--	--	--
Montana	--	--	M	--
Nebraska	I	--	--	--
New Hampshire	--	M	M	M
North Dakota	--	--	M	M
Ohio	M[b]	--	M	--
Oklahoma	I	I	M	M
Puerto Rico	--	M	M	--
South Carolina	--	I	I	I
Tennessee	I	I	I	I
Utah	--	--	--	M
West Virginia	--	--	I	M
Wyoming	I	I	I	I
I = cannabis is illegal				
M = medical cannabis is legal				
R = recreational and medical cannabis are legal				

[a]-- represents the BRFSS cannabis module was not asked in that state in that year
[b]Medical cannabis became legal in Ohio in June 2016

© 2021 Jeffers AM et al. *JAMA Network Open.*

eTable 2. Distribution of Covariates Within Sample (States During Years Where Optional Cannabis Module Was Given, N = 387 157) and Out of Sample (States During Years When Optional Cannabis Module Was Not Given, N = 1 404 866). In-sample and out-of-sample covariates were distributed significantly different at the 95% confidence level except for gender (p=0.65) and marital status (p= 0.64), which were not significantly different between in- and out-of samples. While there are statistically significant differences as shown, most quantifiable differences between sample characteristics are quite small.

	In Sample (Cannabis module given)	Out-of-Sample (Cannabis module not given)	P-value
	%	%	
**Total**	24.5	75.4	
**Age**			
18-34 years	27.9	29.7	<0.001
35-64 years	50.3	49.5	
65+ years	21.8	20.8	
**Gender**			
Male	48.5	48.6	0.65
Female	51.5	51.4	
**Race/Ethnicity**			
White	57.3	65.1	<0.001
African American	9.8	12.6	
Asian, Native Hawaiian, or Pacific Islander	7.6	4.5	
Native American	0.8	1.0	
Hispanic	22.6	15.1	
Other	1.9	1.8	
**Marital Status**			
Not married	49.4	49.5	0.64
Married	50.6	50.5	
**Educational Status**			
≤ High school degree	40.9	41.6	<0.001
Some college	31.8	30.7	
College degree	27.2	27.7	
**Employment Status**			
Employed	56.6	57.4	<0.001
Unemployed	12.0	12.5	
Student	5.4	5.6	
Retiree	19.3	18.6	
Homemaker	6.7	6.0	
**Annual Household Income**			

	In Sample (Cannabis module given)	Out-of-Sample (Cannabis module not given)	P-value
	%	%	
<$25,000	27.0	26.8	<0.001
$25,000 to <$75,000	36.9	38.5	
$75,000+	35.2	34.6	
**Legality of Cannabis in State of Residence**			
No legal medical or recreational cannabis	27.7	40.5	<0.001
Legalized medical cannabis	37.6	48.9	
Legalized recreational cannabis	34.8	10.6	
**Smoking**			
Current smoker	14.4	16.4	<0.001
Former smoker	24.2	24.3	
Never smoker	61.4	59.4	
**E-cigarettes**			
Current e-cigarette user	3.2	3.5	<0.001
Former e-cigarette user	12.4	12.2	
Never e-cigarette user	59.3	57.4	
No e-cigarette question (2019)	25.1	26.9	
**Alcohol**			
Any alcoholic beverages in past 30 days	51.9	53.6	<0.001
Any alcohol binges in past 30 days	15.6	16.9	<0.001

© 2021 Jeffers AM et al. *JAMA Network Open.*

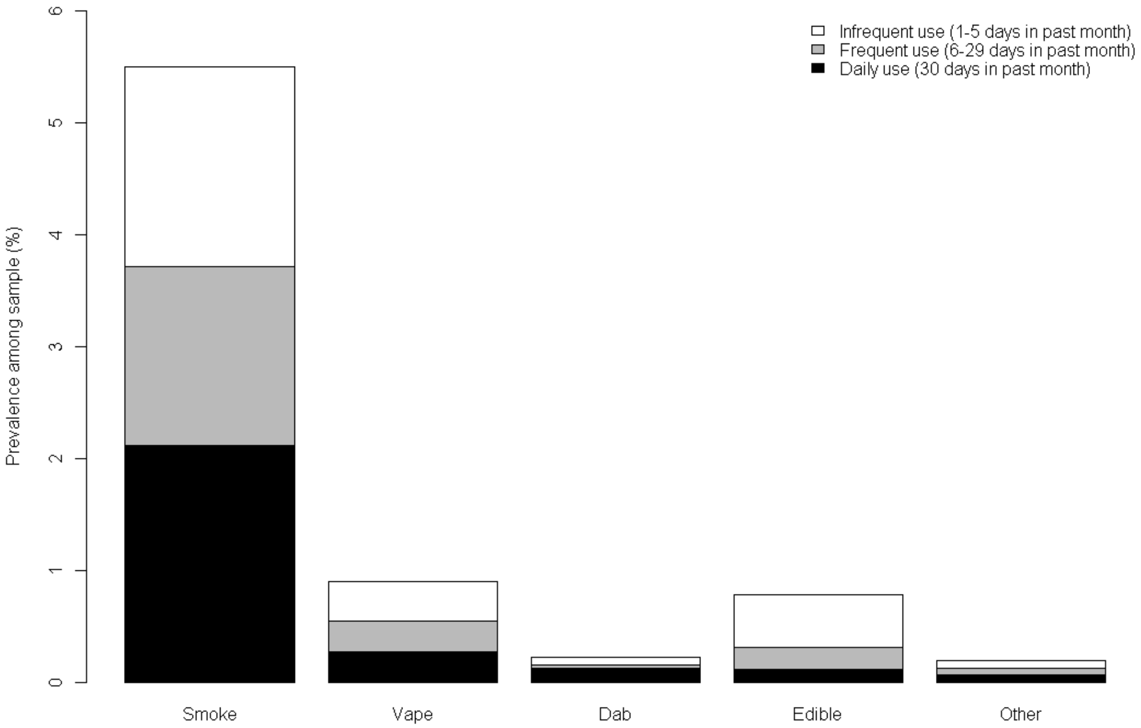

eFigure 1. Prevalence of Prominent Forms of Cannabis Use by Frequency of Use.[a] The majority of cannabis smokers were frequent or daily users (67.5%), while the majority of edible cannabis users were infrequent users (59.6%). The unweighted sample size of those smoking cannabis was 13,901, vaping cannabis was 1,977, dabbing cannabis was 462, using cannabis edibles was 1,974, and other methods of cannabis use was 646.

[a] Does not sum to total prevalence of past-month cannabis use because 25% of cannabis users did not report predominant form of use

© 2021 Jeffers AM et al. *JAMA Network Open.*

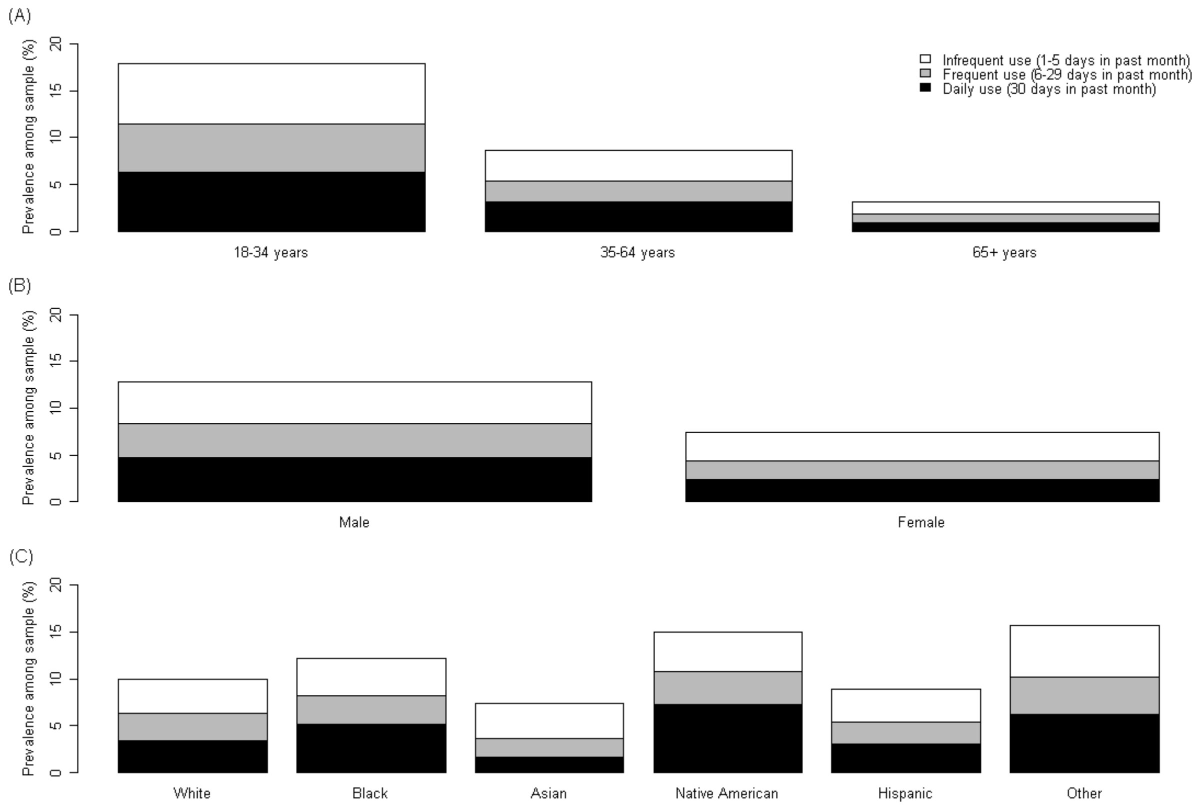

eFigure 2. Prevalence of Cannabis Use Frequency by Age Group, Gender, and Race/Ethnicity.

Panel A: Prevalence of cannabis use frequency by age group. Among cannabis users, close to two-thirds of those ages 18-34 years (64.1%) or ages 35-64 years (62.2%) were frequent or daily cannabis users. Over half (58.7%) of individuals who used cannabis over the age of 65+ engaged in frequent and daily cannabis use. Unweighted sample sizes of subgroups were 58,009 for ages 18-34 years, 186,923 for ages 35-64 years, and 142,225 for ages 65+ years. **Panel B: Prevalence of cannabis use frequency by gender.** Frequent and daily use were more common among male (64.3%) than female (59.2%) cannabis users. Unweighted sample sizes of subgroups were 171,371 for men and 215,647 for women. **Panel C: Prevalence of cannabis use frequency by race/ethnicity.** Frequent and daily cannabis use represented 63.1% of White, 67.1% of Black, 48.9% of Asian, 71.7% of Native American, 60.5% of Hispanic, 64.9% of Other race/ethnicity-identifying cannabis users. Unweighted sample sizes of subgroups were 292,210 for White, 28,345 for Black, 9,582 for Asian, 5,609 for Native American, 36,697 for Hispanic, and 8,401 for Other respondents.

© 2021 Jeffers AM et al. *JAMA Network Open.*

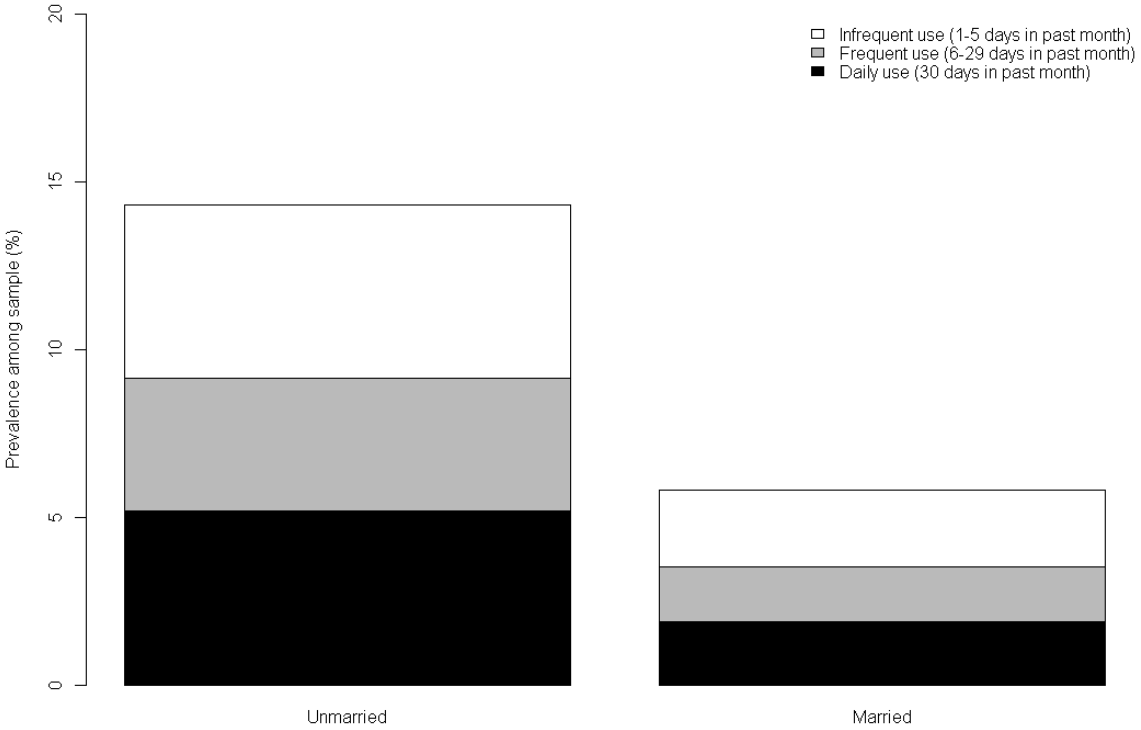

eFigure 3. Prevalence of Cannabis Use Frequency by Marital Status. The proportion of frequent and daily use is slightly greater among unmarried cannabis users (63.8%) compared to married ones (60.8%). The unweighted sample size of unmarried individuals was 181,153 and 203,841 for married individuals.

© 2021 Jeffers AM et al. *JAMA Network Open.*

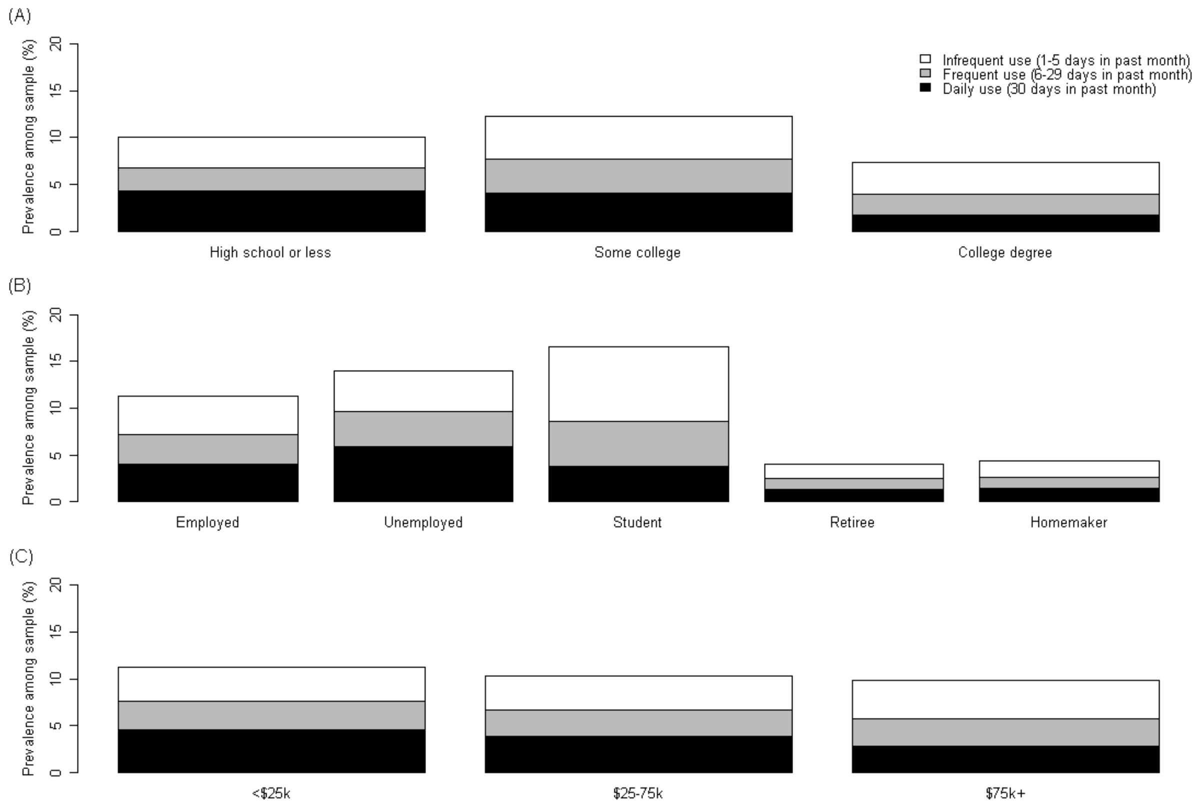

eFigure 4. Prevalence of Cannabis Use Frequency by Educational Attainment, Employment Status, and Annual Income.

Panel A: Prevalence of cannabis use frequency by educational attainment. Among cannabis users, the proportion of frequent and daily use was higher for those with educational attainment of a high school education or less (67.8%) and some college (62.6%), than those with a college degree (54.3%). Unweighted sample sizes were 135,117 for individuals with high school educational attainment or less, 109,676 for some college, and 141,280 for college degrees. **Panel B: Prevalence of cannabis use frequency by employment status.** The proportion of frequent and daily use out of any past month cannabis use was 63.2% for the employed, 68.6% for the unemployed, 51.9% for students, 61.6% for retirees, and 59.9% for homemakers. Unweighted sample sizes for subgroups were 189,876 for the employed, 42,640 for the unemployed, 9,181 for students, 121,805 for retirees and 21,324 for homemakers. **Panel C: Prevalence of cannabis use frequency by annual income.** The proportion of frequent and daily cannabis use out of any past month cannabis use decreased as income increased (<$25,000, 67.7%; $25,000-$74,999 64.5%; $75,000+, 58.0%). Unweighted sample sizes for subgroups were 87,253 for respondents with income <$25,000, 133,456 for incomes $25,000-$74,999, and 108,635 for incomes $75,000+.

© 2021 Jeffers AM et al. *JAMA Network Open.*

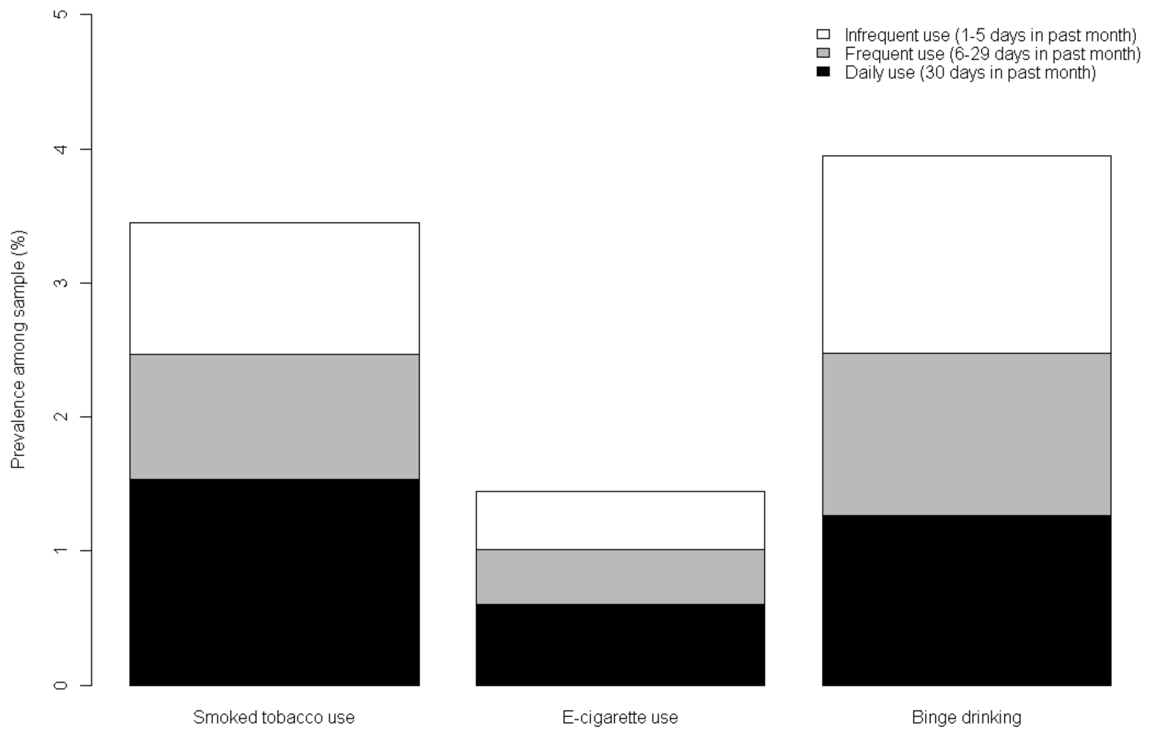

eFigure 5: Prevalence of Dual Use of Cannabis and Smoked Tobacco, e-Cigarettes, and Binge Drinking by Frequency of Cannabis Use. Daily use of cannabis was more common among current tobacco smokers. Over two-thirds of those with dual use of cannabis and either smoked tobacco (71.6%) or e-cigarettes (70.1%) were daily or frequent cannabis users. Over half (62.8%) of dual binge drinkers and cannabis users engaged in frequent and daily use. The unweighted sample size for dual users of smoked tobacco and cannabis was 9,416, dual users of e-cigarettes and cannabis was 2,327, and dual binge drinkers and cannabis users was 9,294.

© 2021 Jeffers AM et al. *JAMA Network Open.*

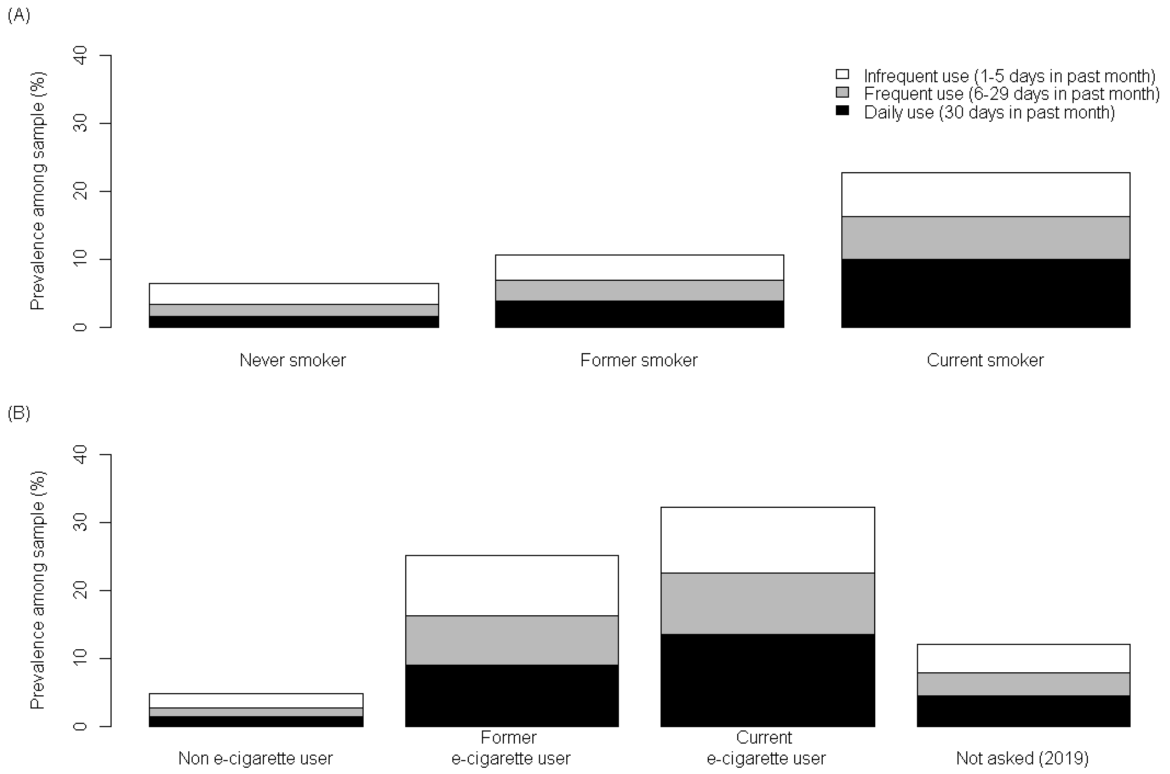

eFigure 6. Prevalence of Cannabis Use Frequency by Tobacco Product Use
Panel A. Prevalence of cannabis use frequency by tobacco smoking status. Among cannabis users that had never smoked tobacco, almost half were infrequent users (46.0%), while cannabis users who currently smoked tobacco were predominantely frequent and daily users (71.6%). The unweighted sample size of non-smokers was 221,775 , former smokers was 108,703, and current smokers was 54,490. **Panel B. Prevalence of cannabis use frequency by e-cigarette use status.** Cannabis users that had never used e-cigarettes were predominately infrequent users (43.8%), while former (64.7%) and current e-cigarette users (70.1%) were predominately frequent and daily cannabis users. The unweighted sample size of non e-cigarette users was 229,643, former users was 35,494, and current e-cigarette users was 9,097. The sample size of those not asked about e-cigarette use (2019) was 90,833.

© 2021 Jeffers AM et al. *JAMA Network Open.*

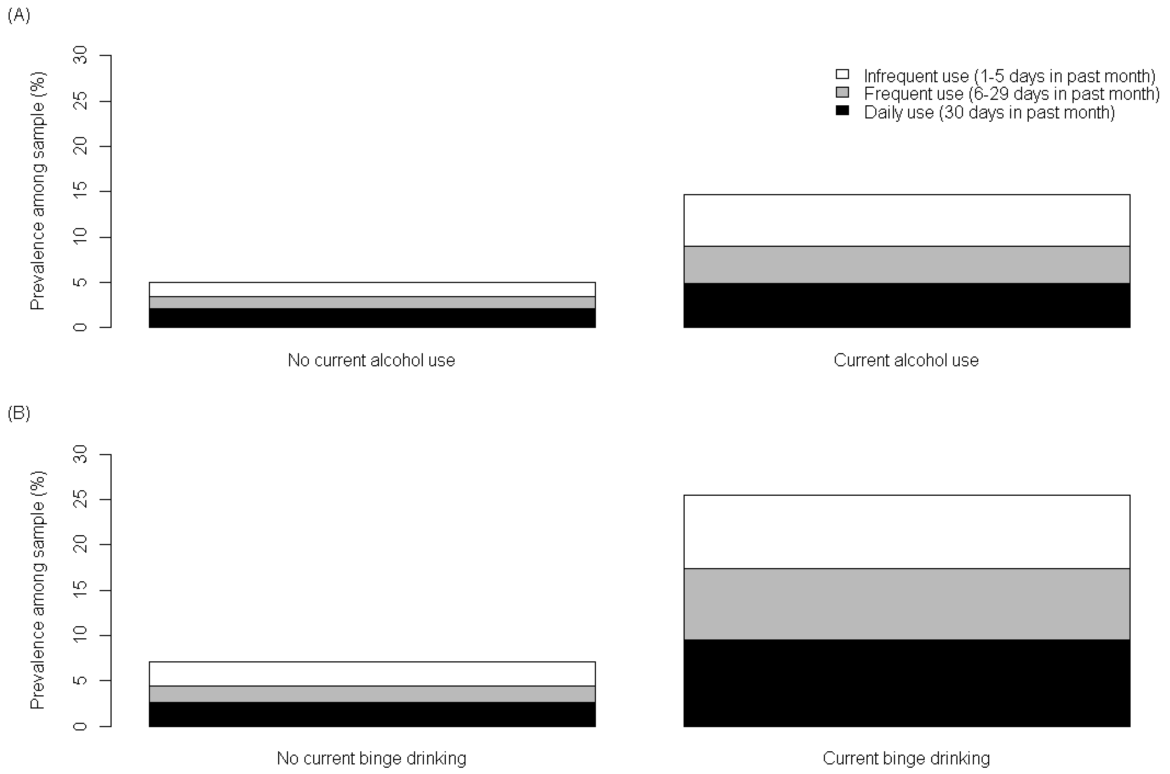

eFigure 7. Prevalence of Cannabis Use Frequency by Alcohol Use

Panel A. Prevalence of cannabis use frequency by past month alcohol use. The proportion of daily cannabis use was higher among non alcohol users (42.2%) than current alcohol users (32.9%). The unweighted sample size of those not using alcohol in the past month was 192,382 and current alcohol use was 190,865. **Panel B. Prevalence of cannabis use frequency by binge drinking status.** Proportion of frequent and daily cannabis use was similar among respondents who had engaged in binge drinking (62.8%) within the past month and those that had not (63.0%). The unweighted sample size of no binge drinking within past month was 332,296 and binge drinking was 48,302.

© 2021 Jeffers AM et al. *JAMA Network Open.*

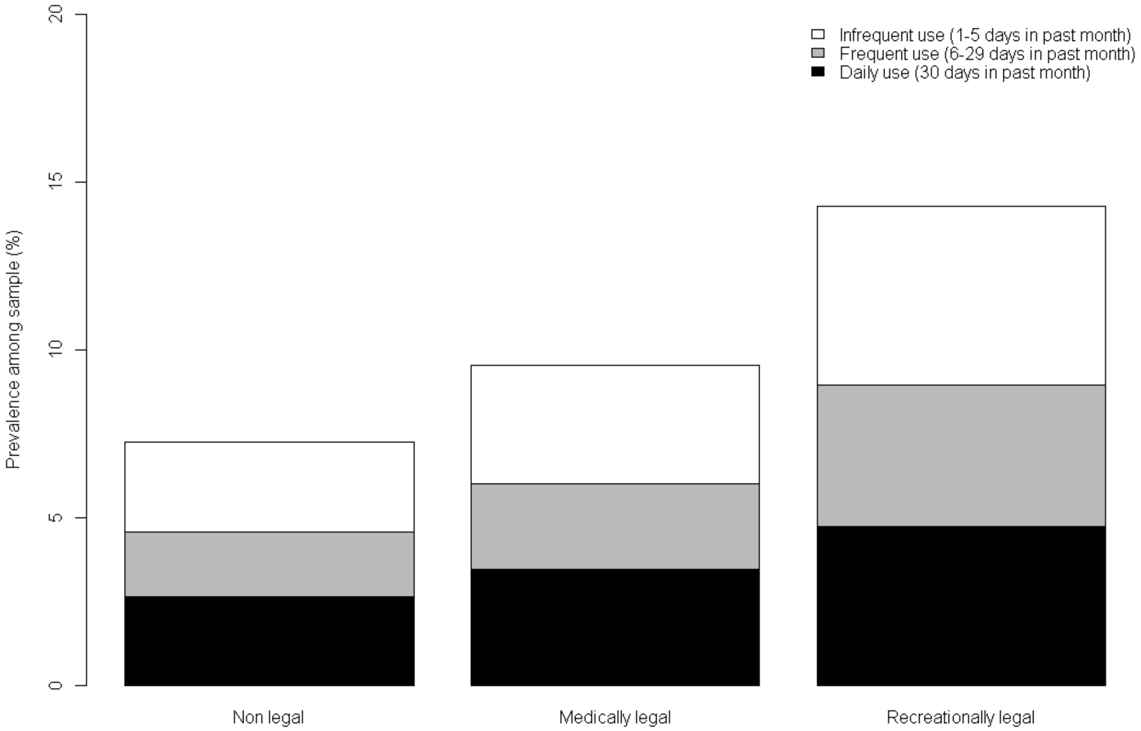

eFigure 8. Prevalence of Cannabis Use Frequency by Legality of Cannabis in State of Residence. The proportion of frequent and daily cannabis use out of any past month cannabis use was similar among those residing in states with non legal cannabis (63.0%), medically legal cannabis (63.0%), and recreationally legal cannabis (62.8%). The unweighted sample size of residents of states with non legal cannabis is 144,366, medically legal cannabis is 197,915, and recreationally legal cannabis is 44,876.

© 2021 Jeffers AM et al. *JAMA Network Open.*
